# Fluorescent dye labeled DNA size standards for molecular mass detection in visible/infrared range

**DOI:** 10.1186/1756-0500-4-12

**Published:** 2011-01-21

**Authors:** Soni Gupta, Chaitanya Charakana, Yellamaraju Sreelakshmi, Rameshwar Sharma

**Affiliations:** 1 Department of Plant Sciences, School of Life Sciences, University of Hyderabad, Hyderabad 500 046, India

## Abstract

**Background:**

Targeting Induced Local Lesions in Genomes (TILLING) is a high throughput reverse genetics tool which detects mismatches (single point mutations or small indels) in large number of individuals of mutagenized populations. Currently, TILLING is intensively used for genomics assisted molecular breeding of several crop plants for desired traits. Most commonly used platform for mutation detection is Li-COR DNA Analyzer, where PCR amplified products treated with single strand mismatch specific nuclease are resolved on denaturing gels. The molecular size of any cut product can be easily estimated by comparing with IR dye labeled markers of known sizes. Similar fluorescent dye labeled size markers are also used for several genotyping experiments. Currently, commercially available size standards are expensive and are restricted up to only 700 bp which renders estimation of products of sizes greater than 700 bases inaccurate.

**Findings:**

A simple protocol was developed for labeling 5' end of multiple DNA size markers with fluorescent dyes. This method involves cloning a pool of different size markers of DNA in a plasmid vector. PCR amplification of plasmid using IR dye labeled universal primers generates 5' fluorescent labeled products of various sizes. The size of products constituting the ladder can be customized as per the need. The generated size markers can be used without any further purification and were found to be stable up to one year at -20°C.

**Conclusions:**

A simple method was developed for generating fluorescent dye labeled size standards. This method can be customized to generate different size standards as per experimental needs. The protocol described can also be adapted for developing labeled size standards for detection on platforms other than Li-COR i.e. other than infra red range of the spectrum.

## Background

Size standards are integral part of any electrophoretic separation to determine the approximate size of the resolved experimental samples. For most applications pertaining to nucleic acids, intercalating dyes (such as ethidium bromide) are used for staining which aids visualization of DNA/RNA during or post-electrophoretic run. In contrast, the detection of PCR amplified DNA products separated on polyacrylamide gels on commercial Analyzers like Li-COR (Li-COR, Nebraska, USA) requires size markers to be labeled with special fluorescent dyes as samples are scanned in the infra-red range of the spectrum during the electrophorectic run. Currently, Li-COR Analyzers are largely used for detection of point mutations by TILLING, which relies on polyacrylamide gel electrophoretic separation of PCR amplified DNA (fluorescently labeled) which is subsequently digested with CEL I endonuclease at the point of mismatch [1,2]. Though, the IR dye labeled markers of different sizes are commercially available, currently the maximum size is restricted to 700 bp. On the other hand, PCR products up to 1.5 Kb can be subjected to TILLING on Li-COR machine. Consequently, non availability of size markers longer than 700 bp makes estimation of the size of large products difficult, which is needed to approximate location of putative mutations in the DNA.

Here we report a protocol to generate fluorescently labeled size standards.

## Methods and Results

The size standards can be generated either by PCR amplification with different sets of primers amplifying varying size products or by cutting a plasmid with restriction endonucleases and end labeling the fragments with a fluorescent tag. We adopted the latter approach with certain modifications. We surpassed the requirement of restriction digestion and used commercially available size standards that were PCR amplified and fluorescently labeled. The latter procedure reduced the cost as well as time needed for designing primers for generation of different size standards. Commercially available 100 bp DNA ladder (Medox Biotech India Pvt. Ltd.) was used as the source of size standards (100-1000 bp) consisting of standards having 100 bp increment in size.

About 2 μg of the DNA was precipitated by adding 1/25^th ^volume of 5 M NaCl and 2 volumes of cold ethanol and incubated at -20°C for 2 hours. The DNA was pelleted at 12,000 rpm for 15 min at 4°C followed by 70% ethanol wash. The pellet was air dried and dissolved in 10 μl sterile water. Addition of deoxyadenosine residues at the 3' ends of the size standards was done prior to cloning. The standards were incubated with 1 μl of 10 mM dATP, 1.5 μl of 10× PCR buffer (100 mM Tris, 500 mM KCl, 15 mM MgCl_2_, 0.1% (w/v) gelatin, 0.05% (v/v) Tween-20, 0.05% (v/v) NP-40, pH 8.8), 0.20 μl Taq DNA polymerase (in-house isolated) in a total volume of 15 μl at 72°C for 15 min. The pool of DNA size standards carrying A at their 3' ends were ligated to pGEM^®^-T Easy Vector (3015 bp, Promega) carrying M13 forward and reverse sequencing primer binding sites. The ligated product was transformed in ultra-competent *E. coli *DH5α cells followed by plating on Luria Bertani agar plates supplemented with 50 μg/ml ampicillin and incubated at 37°C overnight. Transformants were analyzed by colony PCR using M13F (TGTAAAACGACGGCCAGT) and M13R (AGGAAACAGCTATGACCAT) primers. Plasmid DNA was isolated from the positive clones by alkali lysis method and purified by PEG 8000. The pellet was air-dried and dissolved in 20 μl sterile water.

The plasmids having the desired inserts were amplified with unlabeled M13 forward and reverse primers. The PCR reaction contained 20-25 ng of plasmid DNA, 1× PCR buffer, 200 μM dNTPs, 3 pmoles each of M13 forward and reverse primers (unlabeled) and 0.18 μl of Taq DNA polymerase in a total volume of 20 μl. After confirmation of PCR amplification on agarose gel, the products were 2-fold diluted with sterile water. PCR reaction with labeled M13F and M13R primers (MWG) was carried out on individual amplified products in two sets. The first set of PCR reaction contained about 1 μl of the diluted amplified product, 1× PCR buffer, 200 μM dNTPs, 1 pmole each of M13 forward primer (7:3 ratio of IR dye labeled and unlabeled), M13 reverse primer (unlabeled) and 0.18 μl of Taq DNA polymerase. The second set PCR was similar as described above except that M13 forward primer (unlabeled) and M13 reverse primers (7:3 ratio of IR dye labeled and unlabeled) were used. The cycling conditions were 94°C for 4 min; 35 cycles of 94°C for 20 sec, 60°C for 30 sec, 72°C for 1.5 min; 72°C for 10 min followed by 4°C forever. 5 μl aliquots from each reaction were mixed with 2 μl formamide loading buffer {37% (v/v) deionized formamide, 1 mM EDTA and 0.02% (w/v) bromophenol blue}. To generate a cocktail of different size standards, an aliquot from all of the above PCR amplifications were pooled for usage as labeled size standard for Li-COR without any further purification. The cocktails of above products (0.3-0.5 μl) was electrophoresed on 6.5% (w/v) denaturing polyacrylamide gel in 0.8% TBE buffer at 1500 V, 40 mA and 40 V setting on 4300 Li-COR Analyzer for 4 hours. The TIFF images of 700 and 800 channels respectively, were analyzed in Adobe Photoshop software (Adobe Systems Inc.).

**Figure 1:**
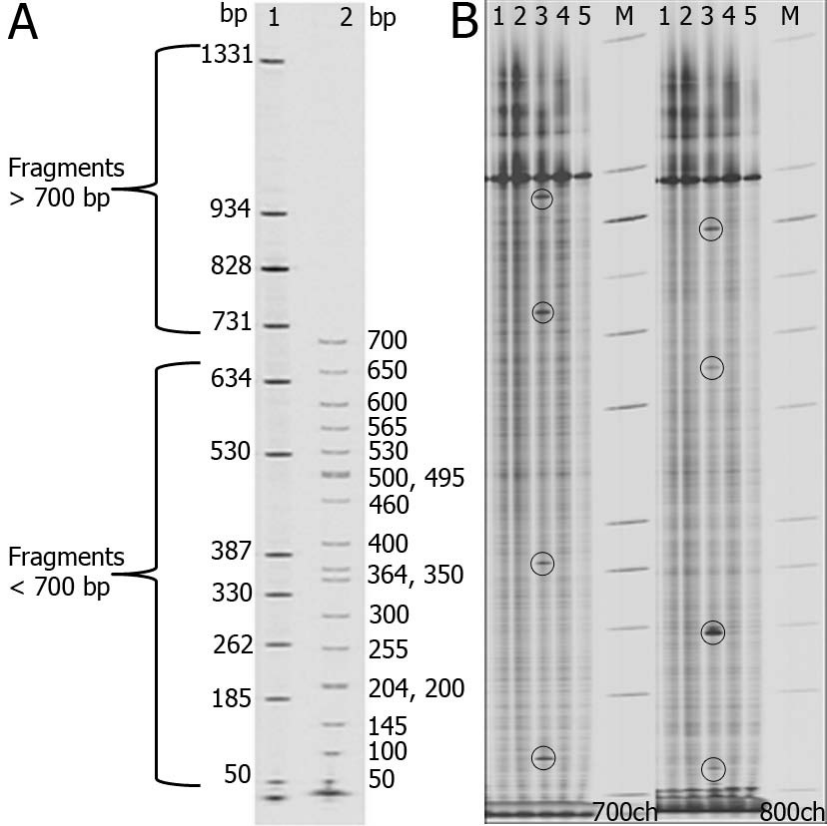
**The dye labeled size standards for molecular mass determination.** A) IR dye labeled size standards for Li-COR Analyzers. Comparison of custom generated size standards with the commercially available size standard from Li-COR Biosciences. The numbers on left and right side of gel picture refer to size of fragments in bp respectively. Lane 1 - Custom generated size standards, Lane 2 - Li-COR size standards. B) Customized size standards used for SNP detection. Images of Li-COR gel showing resolved size standards in 700 and 800 channels. One of the samples having multiple SNPs (demarcated as black circles in the image) is shown. The samples were amplified with IR dye labeled gene specific primers for *LeACS-2*. A full-length fragment of 928 bases was obtained. PCR amplification, CEL I digestion and electrophoresis on polyacrylamide gel was done as previously described by Till *et. al*., 2006 [2]. Lanes 1, 2, 3, 4, 5 - DNA samples from different tomato accessions doped with reference DNA (Arka Vikas); Lane M - customized size standards.

To determine the exact molecular size of the standards generated using M13 forward and reverse unlabeled primers, the PCR products were sequenced by ABI Big dye terminator chemistry (*v *3.0) {Applied Biosystems}. Figure 1A shows the molecular size of the generated standards in the ladder and its comparison with the commercially available size standards from Li-COR. Figure 1B shows a part of the polyacrylamide gel with in-house generated size standards loaded adjacent to mutant DNA samples showing the complementary cut products in the 700 and 800 channels. One of the advantages of custom labeled standards is that these can be used to demarcate the left and right sides of gel. For this, two different cocktails of size standards can be made by mixing individual standards. The left side markers can contain all the standards whereas the right side markers can have just few of the standards. Employing two sets of standards can eliminate any error that may occur due to accidental flipping of paper combs. Using two different cocktails the left and right side of the gel can be visually detected during electrophoresis. In addition, a single size standard such as of 200 or 300 bp size can be loaded in every 8^th ^lane to aid precise identification of lane with an increment of 8 numbers during mutation/SNP analysis.

Occasionally some faint spurious bands may appear along with the size standards on Li-COR. Primarily these bands arise due to some degree of mispriming during PCR amplifications with M13 forward and reverse primers. Though these bands are undetectable on agarose gels but owing to the sensitive nature of laser detection are visible during run on Li-COR machine. In such case, the given size standard can be diluted to a level allowing only the standard of interest to be visible on Li-COR machine.

## Conclusions

We have developed a simple method for generating IR dye labeled size standards for usage on Li-COR DNA Analyzers which scans samples using two inbuilt diode lasers that operates at 685 and 785 nm, respectively. Our protocol uses fluorescently labeled universal primers that are often used to reduce the cost of mutation detection. The choice of sizes for different standards to generate a cocktail can be customized for genotyping or TILLING protocols. Though the above method basically generates labeled standards for Li-COR Analyzers, it can also be easily adapted to other detection systems requiring labeled size standards.

## Competing interests

The authors declare that they have no competing interests.

## Authors' contributions

SG has conceptualized and executed the work. CC has helped in plasmid DNA isolation. SG, YS and RS wrote the manuscript. All authors read and approved the manuscript.
